# Unusual Sequential Development of Multiple Sclerosis, Thymoma, Myasthenia, and Lymphoma in the Same Patient

**DOI:** 10.1155/carm/4552045

**Published:** 2026-07-29

**Authors:** Josef Finsterer

**Affiliations:** ^1^ Neurology Neurophysiology Center, Vienna, Austria

**Keywords:** interferon, lymphoma, multiple sclerosis, myasthenia, thymoma

## Abstract

No case has yet been described in which a patient developed multiple sclerosis (MS) followed by thymoma, myasthenia gravis (MG), and T‐cell lymphoma. The patient is a 47‐year‐old woman who developed relapsing‐remitting MS at the age of 31 and was treated with interferon‐beta (IFN‐β) for over seven years. She was then diagnosed with thymoma, which was treated with chemotherapy and surgery. One year later, she was diagnosed with MG, which was treated with steroids, pyridostigmine, and intravenous immunoglobulins. Due to an MS relapse at the age of 40, ocrelizumab was administered for 2.5 years, after which she developed T‐cell lymphoma. As the lymphoma had responded only partially to chemotherapy, an autologous stem cell transplantation was performed. It remains unclear whether there was a causal link between the immunosuppressive MS treatment and the thymoma or lymphoma, whether these developed due to underlying immune dysregulation, individual predisposition, or the cumulative effect of prior oncological therapies, or whether this was merely a chance co‐occurrence of rare diseases. The case shows that a single patient may sequentially develop MS, MG, thymoma, and T‐cell lymphoma. Fundamental and comprehensive clinical studies are required to elucidate the underlying pathophysiology and to confirm or refute a possible causal link.

## 1. Introduction

Relapsing‐remitting multiple sclerosis (MS, RRMS) is a common immunological disease that responds well to immunosuppressive therapy [1]. The underlying pathophysiology is characterized by a breakdown of the blood–brain barrier, which allows B‐lymphocytes to infiltrate and mistakenly attack the myelin sheath and axons. Only in rare cases is immunosuppressive treatment complicated by side effects of varying severity [2]. Malignancies are one of these rare complications of immunosuppressive drugs [3]. The most common tumors following immunosuppression include skin cancer, cervical cancer, and visceral carcinomas [4]. Thymomas or lymphomas following the use of immunosuppressive drugs for the treatment of MS have hardly been reported [5]. Thymomas are known for their association with myasthenia gravis (MG) [6]. MG is usually diagnosed before the discovery of a thymoma, but only 15% of MG patients have a thymoma, while half of the patients with thymoma develop MG [6]. Whether causal or pathophysiological links exist between MS, thymomas, and lymphomas has been little investigated. To the best of our knowledge, a patient who sequentially developed MS, a thymoma, MG, and a T‐cell lymphoma has not previously been reported. The following report details such a case and discusses the potential underlying pathophysiological mechanisms and whether causal links exist.

## 2. Case Report

The patient is a 47‐year‐old woman, height 168 cm, weight 64 kg, who, at the age of 31, developed neck stiffness, dizziness, double vision when moving her hands quickly, and difficulty writing with her right hand, 6 weeks before the in‐hospital assessment. One day before admission, she additionally developed weakness of the left side of her face and, a few days earlier, coordination problems in her right leg. The neurological examination revealed a positive Lhermitte’s sign, paralysis of the left side of her face, double vision when looking to the left, gaze‐evoked nystagmus, and fine motor impairment of her right hand. MRI of the brain showed > 10 supratentorial and > 10 infratentorial lesions (left occipital [contrast‐enhancing], periventricular, bilateral in the cerebellar hemispheres (contrast‐enhancing), in the medulla, and in the myelon at C2 [contrast‐enhancing]). An examination of the cerebrospinal fluid (CSF) revealed autochthonous IgG production and positive oligoclonal bands. The diagnosis of MS was made based on the clinical presentation, CSF findings, and positive Barkhof/Tintore criteria. Steroids were administered with a positive effect, followed by beta interferon (IFN‐β). Despite this treatment, she suffered a relapse 4 months later, which manifested itself in paralysis of the right side of her face and numbness in her left thigh and right face. A second examination of the CSF revealed no change from the previous findings. Steroids were administered to treat the relapse.

At the age of 38, a small cell thymoma (WHO ypT3, ypN0, ypM1a, L1, V0, R0, and B3) was accidentally discovered in the left chest wall and treated with adjuvant chemotherapy prior to pleuropneumonectomy, partial resection of the diaphragm and pericardium, and diaphragmatic and pericardial plasty. The Karnofsky index was 90%. Consequently, treatment with IFN‐β was discontinued. No thymoma recurrence occurred during the following 5 years.

At the age of 39, the patient developed successive episodes of exertion‐induced double vision, ptosis, transient head drop, and, four months later, evening dysarthria that resolved completely by the following morning. Four months later, she also developed dysphagia, which likewise worsened in the evening. The examination of these symptoms 1 month later revealed a positive Simpson test, elevated antibodies against muscular acetylcholine receptors to 85 nmol/L (*n*, < 0.4 nmol/L), and a 12% decrease after low‐frequency repetitive nerve stimulation of the facial and accessory nerves. A recurrence of the thymoma was ruled out. MG was diagnosed and treatment with pyridostigmine (320 mg/day), and prednisolone was initiated. Under this therapy, the symptoms gradually improved and disappeared completely a few weeks later. Given the history of thymoma, azathioprine was avoided, and intravenous immunoglobulin (IVIG) (20–30 g/month) was administered instead.

At age 40, a follow‐up MRI of the brain because of fatigue showed two new MS plaques, and a second MRI 5 months later showed several new lesions. For this reason, it was decided to start treatment with ocrelizumab. In preparation for this B‐cell‐depleting therapy, the patient received a pneumococcal vaccination, after which she developed a myasthenic crisis. After the first ocrelizumab infusions (300 mg twice within three weeks), she developed a second myasthenic crisis. Despite these events, she received a second dose of ocrelizumab (600 mg) and a third cycle (600 mg) four months later. Lymphocyte typing after ocrelizumab showed a CD19/CD20 ratio of 4.1% of lymphocytes and 55 CD19 cells per microliter. No further cerebral lesions occurred after this treatment.

At a regular follow‐up examination at the age of 44 using chest CT revealed an increase in scar tissue at the thymoma resection site. A biopsy of this lesion confirmed a recurrence of the thymoma. Staging using FDG‐PET revealed bone metastases in T6 and in the ribs on the left side. The recurrence was treated with radiation therapy. Ocrelizumab was discontinued.

At the age of 45, she suffered a herpes zoster infection in the right dermatomes C2‐3. Due to persistent leukopenia, a bone marrow biopsy was also performed, but no abnormalities were found. However, a second bone marrow biopsy 3 months after the first revealed a T‐cell receptor gamma/delta‐positive lymphoma with mutations in TET2, ETV6, BCOR, and RPS6KA4. PET‐CT showed FDG‐avid soft tissue formation of the ventrolateral left chest wall and suspicious lymph nodes, hepatosplenomegaly, and bone metastases in T6 and the left ribs without FDG avidity. The hepatosplenic lymphoma was treated with splenectomy and four cycles of the dexamethasone, methotrexate, ifosfamide, *Escherichia coli* L‐asparaginase, and etoposide (SMILE) regimen. The follow‐up PET‐CT showed a slight reduction in size, a marked reduction in contrast agent accumulation, but unchanged bone lesions. The PET‐CT 4 months later again showed an enlargement of the soft tissue formation. The bone marrow biopsy again showed bone marrow infiltration by T‐cell lymphoma. The relapse was treated with two cycles of ifosfamide, carboplatin, and etoposide (ICE) [1]. A subsequent PET‐CT showed a slightly progressive FDG‐avid lesions in the left chest wall, a non‐FDG‐avid lesion in the left internal mammary group that was decreasing in size, and non‐FDG‐avid lesions in the bones that were constant in size. Since the treatments used were ineffective, it was decided to perform an autologous stem cell transplant (ASCT). Prior to ASCT, she underwent high‐dose chemotherapy with bendamustine, etoposide, cytarabine, and melphalan (Benda‐EAM). Compared to the previous examination, the MRI of the brain and spinal cord showed an unchanged lesion load with no activity (Figure 1). There was no evidence of cerebral lymphoma. The family history was unremarkable regarding immunological disorders; only one aunt had suffered from breast cancer. Her current medication includes buprenorphine, fentanyl, prednisolone (12.5 mg/day), and pyridostigmine.

**FIGURE 1:**
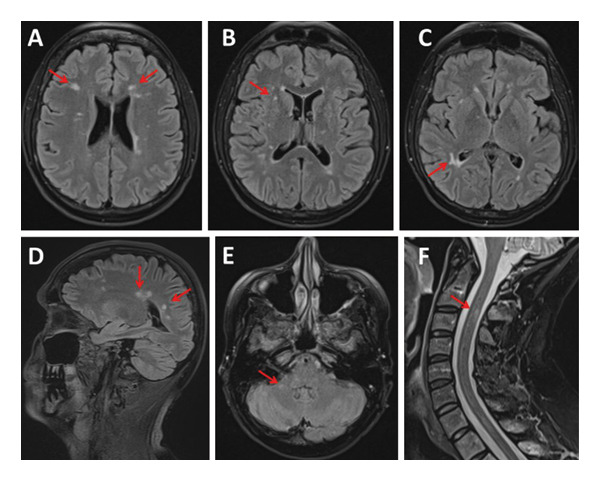
A follow‐up MRI at age 47 revealed a pronounced supratentorial and bihemispheric lesion load, predominantly in the periventricular regions of the lateral ventricles. Additionally, subcortical and juxtacortical lesions measuring up to 8 mm in diameter were observed (Figures (A)–(D) axial and sagittal FLAIR sequences). Furthermore, isolated infratentorial and cerebellar lesions measuring up to 6 mm in diameter were present (Figure (E), axial FLAIR sequence). None of these lesions showed contrast enhancement on T1‐weighted imaging. There was no evidence of blood–brain barrier disruption. A T2‐hyperintense lesion measuring 1 mm in diameter was identified in the cervical spinal cord at the C2/3 level; this lesion also did not show enhancement (Figure (F)).

## 3. Discussion

The patient presented here is interesting in several respects. The patient developed a previously undescribed combination of two autoimmune diseases and two malignant tumors. After being diagnosed with MS and undergoing 7 years of treatment with IFN‐β, the patient developed a thymoma and, 1 year later, MG. Due to an MS relapse, treatment was switched from IFN‐β to ocrelizumab, and after 2.5 years of ocrelizumab therapy and 15 years after the onset of MS, a T‐cell lymphoma was diagnosed.

A causal relationship between IFN‐β and thymoma, respectively, MG has not yet been proven, but there is some evidence from previous studies that IFN‐β actually promotes the development of thymoma. A cell‐based study showed that IFN‐β increases the expression of the chemokines CXCL13 and CCL21 in thymic epithelial cells (TECs) and lymphatic endothelial cells, respectively [7]. These two chemokines are involved in the development of the germinal center and are overexpressed in the MG thymus with follicular hyperplasia [7]. It has also been found that IFN‐β induces the B‐cell activating factor (BAFF), which promotes autoreactive B‐cells and is overexpressed in the TECs of the MG thymus [7]. Furthermore, chronic activation of the IFN‐β signaling pathway can lead to thymic atrophy and impair T‐cell development and tolerance. In a 12‐year long‐term study of 5146 RRMS cases treated with IFN‐β, an increased risk of breast cancer was observed [8]. The role of neutralizing antibodies against IFN‐β, which develop during long‐term use, in the development of cancer is still unclear. Neutralizing antibodies can not only reduce the effect of IFN‐β but also prevent the immune system from controlling tumor growth and spread [9].

Another interesting aspect of this case is that MG developed 1 year after the discovery of the thymoma. Whether a causal link existed between the thymoma and the MG remains speculative. However, it is known that half of patients with thymoma develop MG [6]. The underlying mechanism determining why one patient develops MG while another does not is unknown. Likewise, it remains unclear whether adjuvant chemotherapy prior to thymoma resection played a pathophysiological role in the development of MG. Chemotherapy agents are known to worsen MG and can even trigger myasthenic crises. Immune checkpoint inhibitors are known to be capable of inducing MG [10]. It is also conceivable that the specific histological subtype of the thymoma may have contributed to the development of MG.

A third interesting aspect is that a lymphoma developed 5 years after the start of treatment with ocrelizumab. Ocrelizumab is an established and effective immunosuppressant for severe MS [11]. It is generally well tolerated, but side effects may occur in individual cases. With regard to the risk of malignancy, there are individual studies that report a link between ocrelizumab and breast cancer [12, 13]. In a study of 5680 MS patients receiving ocrelizumab with 18,218 patient‐years of ocrelizumab exposure, the rate of malignancies was 0.46% [14]. In a study of 305 MS patients receiving ocrelizumab, only one developed metastasizing carcinoma during follow‐up [15]. Based on the experience after market launch, breast cancer has developed in 431 women out of 233,196 MS patients who received ocrelizumab. No association between ocrelizumab and lymphomas has been reported to date. The mechanism by which ocrelizumab may induce malignant transformation of cells is unknown but is explained by the immunosuppressive effect of the drug. It is hypothesized that B‐cell inhibitors such as ocrelizumab inhibit the potentially protective role of B‐cells [16]. Furthermore, it cannot be ruled out that hereditary immune dysregulation, chemotherapy for the thymoma, radiotherapy for thymoma recurrence, and the long‐term use of IVIG may have played an additional role in the development of the lymphoma. Since malignancies are usually multifactorial and develop due to genetic predisposition, personality type, life events, lifestyle, and environmental influences [17], not only medications but also additional factors may have led to the occurrence of the malignancies.

Another interesting aspect is that the first use of ocrelizumab in the index patient triggered a myasthenic crisis. No worsening of MG or triggering of a myasthenic crisis by ocrelizumab has been reported to date. On the contrary, ocrelizumab was used to successfully treat MUSK‐positive MG in a 35‐year‐old man who showed no side effects during an eight‐month follow‐up period [18]. The underlying pathophysiology remains to be fully elucidated; however, it is assumed that the B‐cells depleted by ocrelizumab are the same cells responsible for producing protective antibodies that prevent the body from mistakenly attacking its own motor endplates.

The study’s limitations include the inability to establish a causal link based on a single case, the lack of more comprehensive comparative data, the confounding effects of multiple prior treatments, and the absence of genetic testing for underlying congenital immunodeficiencies.

In summary, the case presented suggests that long‐term IFN‐β therapy for MS may be followed by thymoma, that a thymoma may be followed by MG, that ocrelizumab can exacerbate MG, and that ocrelizumab therapy may be followed by lymphoma. Whether a causal link exists between IFN‐β and thymoma, or between ocrelizumab and lymphoma, remains speculative. Equally plausible explanations include underlying immune dysregulation, individual predisposition, cumulative effects of prior oncological treatments, or simply coincidental occurrence of rare diseases. Fundamental and comprehensive clinical studies are required to establish or refute a causal link between IFN‐β and thymoma, as well as between ocrelizumab and lymphoma.

## Author Contributions

Josef Finsterer drafted the case report, helped with draft revision, and reviewed the manuscript critically for important intellectual content and grammatical edits.

## Funding

This study was not supported by any sponsor or funder.

## Disclosure

The author has read and agreed to the published version of the manuscript.

## Ethics Statement

This study was conducted in accordance with the Declaration of Helsinki.

## Consent

The patient gave written informed consent for the publication of this case report and accompanying images.

## Conflicts of Interest

The author declares no conflicts of interest.

## Data Availability

All the data used to support the findings of this case are available as part of the article and references. Further inquiries can be directed to the corresponding author.
